# The Book World of Medicine and Science

**Published:** 1906-06-02

**Authors:** 


### The Book World of Medicine and Science.

Biographic Clinics, Vol. III. George M. Gould, M.D. (Rebman. Pp. 500.)

This untiring writer has published still another book, the burden of which is the manifold troubles which follow defects of vision. He believes that not only frontal headache, but migraine is almost entirely due to abnormal sight, and considers it a fact that any medical man can prove for himself. Extracts are given from the writings of that cultured and artistic writer, John Addington Symonds, to show how this painful affection rendered his life a misery, so much so that apparently he even at times contemplated suicide. All this misery, it is considered, could have been abolished by suitable glasses. The book not only deals with headache and lateral curvature, which is also considered to be produced by endeavours to get the best view of written work, but has articles on right-handedness and left-handedness. Dr. Gould believes—and in this we cannot but agree with him—that the attempt to teach children to use both hands equally well is not only a useless but a harmful fad. Ninety-eight per cent. of children are born with a natural preference for the right hand, which means, of course, that the left side of the brain has a greater inherent predisposition towards directing refinements of movement than the right. Dr. Gould has known a naturally left-handed gentleman handicapped throughout life by being compelled to use his right hand for writing. When writing he was unable to think, owing, no doubt, to the fact that most of his thoughts, so closely dependent upon speech and hearing, were most intimately connected with mental processes taking place on the opposite side of the brain to that from which nervous stimuli, giving rise to the movements of writing, were proceeding. The connection of right-handedness with eyesight may not at once be obvious. Dr. Gould considers, however, that we are not only right-handed, but right-eyed. He thinks, for example, a man puts a gun to the right shoulder because he is right-eyed. The book is interesting and suggestive, and contains also, as may be gathered from the above, much of great practical importance, presented in a readable way.

The Treatment of Gonorrhœa in the Male. By Charles Leedham-Green, M.B., F.R.C.S. (London: Baillière, Tindall, and Cox. 1906. Pp. 151. Price 5s. net.)

Mr. Leedham-Green is to be congratulated on the concise and yet comprehensive manner in which he has dealt with the treatment of gonorrhœa in the male, and its numerous complications. So far as the text goes, it could hardly show sounder judgment or be more clearly expressed; and beyond this there is little scope for criticism. Mr. Leedham-Green gives considerable prominence to chronic prostatis as a frequent complication of gonorrhœa, and he regards it as being, perhaps, the chief factor in keeping up chronic urethritis. The diagnosis, he maintains, can be made only by mocroscopical examination of the prostatic secretion. For the treatment of chronic prostatitis he recommends massage of the prostate *per rectum*, followed by astringent urethral injections. But he gives a necessary note of warning against the danger of converting the patient, by too prolonged local treatment, into a confirmed sexual neurasthenic. Stricture of the urethra has been omitted on the ground that it is too large a subject for the space available, and gonorrhœal ophthalmia has been ignored because it "belongs more correctly to the domain of the ophthalmic surgeon." This plea is difficult to accept, in the first place, because prophylaxis, which is the most important treatment of all, is not in the hands of ophthalmic surgeons; and secondly, because there are numerous practitioners who have no chance of handing over their cases of gonorrhœal ophthalmia to a specialist. In these circumstances, and bearing in mind the fact that gonorrhœal ophthalmia is probably the commonest cause of blindness at the present day, we hope that Mr. Leedham-Green will remedy the omission in the next edition of this book.

### BOOKS RECEIVED.

Mellins Food, Ltd.

"The Care of Infants."

W. B. Saunders and Co.

"Nursing in the Acute Infectious Diseases," by G. P. Paul, M.D.

Henry Kimpton.

"The Mother's Guide to the Feeding and Rearing of Children," 3rd edition, by T. Dutton, M.D.

J. B. Lippincott Co.

"International Clinics," Sixteenth Series, Vol. 1.

Baillière, Tindall, and Cox.

